# Investigation of photocatalytic degradation of phenol by Fe(III)-doped TiO_2_ and TiO_2_ nanoparticles

**DOI:** 10.1186/2052-336X-12-101

**Published:** 2014-06-30

**Authors:** Saeedeh Hemmati Borji, Simin Nasseri, Amir Hossein Mahvi, Ramin Nabizadeh, Amir Hossein Javadi

**Affiliations:** 1 Department of Environmental Health Engineering, School of Public Health and Center for Water Quality Research (CWQR), Institute for Environmental Research (IER), Tehran University of Medical Sciences, Tehran, Iran; 2 Department of Environmental Health Engineering, Tehran University of Medical Sciences, Tehran, Iran; 3 Nanotechnology department, Engineering Research Institute, Tehran, Iran

**Keywords:** Aqueous solution, Phenol, Fe (III)-doped TiO_2_, P25 TiO_2_, Sol–gel method

## Abstract

In this study Fe (III)-doped TiO_2_ nanoparticles were synthesized by sol–gel method at two atomic ratio of Fe/Ti, 0.006 and 0.034 percent. Then the photoactivity of them was investigated on degradation of phenol under UV (<380 nm) irradiation and visible light (>380 nm). Results showed that at appropriate atomic ratio of Fe to Ti (% 0.034) photoactivity of Fe(III)–doped TiO_2_ nanoparticles increased. In addition, the effects of various operational parameters on photocatalytic degradation, such as pH, initial concentration of phenol and amount of photocatalyst were examined and optimized. At all different initial concentration, highest degradation efficiency occurred at pH = 3 and 0.5 g/L Fe(III)–doped TiO_2_ dosage. With increase in initial concentration of phenol, photocatalytic degradation efficiency decreased. Photoactivity of Fe (III)-doped TiO_2_ under UV irradiation and visible light at optimal condition (pH = 3 and catalyst dosage = and 0.5 g/L) was compared with P25 TiO_2_ nanoparticles. Results showed that photoactivity of Fe(III)-doped TiO_2_ under visible light was more than P25 TiO_2_ photoactivity, but it was less than P25 TiO_2_ photoactivity under UV irradiation. Also efficiency of UV irradiation alone and amount of phenol adsorption on Fe(III)-doped TiO_2_ at dark condition was investigated.

## Introduction

Phenolic compounds constitute an important group of wastewater pollutants produced by chemical, petrochemical, paint, textile, pesticide plants, food–processing and biotechnological industries [1]. As the phenolic compounds toxicity is an important problem, their concentration unfortunately prevents of micro-organisms activity in biological wastewater treatment plant. Therefore, the presence of phenols strongly reduces the biological biodegradation of the other components [2]. However some of the most conventional technologies for phenolic compounds degradation such as granular activated carbon (GAC) adsorption and biological treatment are effective in water treatment but they are slow processes and at higher concentrations of the organic contaminants, they present some difficulties during the operation [2]. So now applying of various advanced techniques in the fields of environmental protection has become prevalent.

Photoassisted catalytic decomposition of aqueous and gaseous contaminants by application of semiconductors as photocatalysts is one of the promising technologies [3,4]. Among various oxide semiconductor photocatalysts, titanium dioxide has been proved to be the most suitable catalyst for widespread environmental applications, considering its biological and chemical inertness, strong oxidizing power, non–toxicity, insolubility, comparatively low cost and long term stability against photo corrosion and chemical corrosion [4-6]. The photocatalytic activity of semiconductor is the result of the production of excited electrons in its conduction band, along with corresponding positive holes in the valence band under UV illumination [5], that react with contaminants adsorbed on the photocatalyst surface [4]. However, the relatively large band gap of TiO_2_ (3.2 eV) limits the efficiency of photocatalytic reactions due to high recombination rate of photogenerated electrons and holes formed in photocatalytic process and low absorption capability of visible light [7]. In this respect, strategies may be suggested to electron–hole recombination rate reduction and photocatalyst efficiency increase [1]. Also shifting the absorption edge to larger wavelengths by adding dopants (metal ions or non-metal) to TiO_2_, while keeping a good control of the main particle size to produce nanoscale configurations of the catalysts can be considered [1,5,8].

Doping TiO_2_ with transition metal cations is an efficient strategy to reduce electron–hole recombination rate and increase photocatalytical efficiency [8]. Noble metals such as Pt are most studied, and other metals such as Au, Pd, Ru, and Fe have been reported to be useful for photocatalytic reactions [4]. Among these various metal ions, Fe(III) has been proved to be a successful doping element [5,7,8] where its radii (0.64 Å) is similar to that of Ti(IV) (0.68 Å), hence Fe(III) will easily substitute Ti(IV) into the lattices of TiO_2_. As Fe(II)/Fe(III) energy level lies close to that of Ti(III)/Ti(IV), Fe(III) can provide a shallow trap for photo-generated hole and electron in anatase. Illuminating Fe(III) can enhance the photogenerated electron–hole pair separation and quantum yield [4,9]. Consequenly the doping technique seems to be one of the most important factors for controlling the reactivity of Fe(III)-doped titania [10].

Among many existing preparation methods, sol–gel is widely used to prepare metal ion doped TiO_2_ due to its flexibility to control pore structures and dopant concentration, and high level of chemical purity [5]. The role of iron ions in TiO_2_ lattice have been discussed extensively in the literature [4,7,8,11-13]. Fe(III) ions can act as electron and hole trappers to reduce the photo-generated hole–electron recombination rate and enhance the photocatalytic activity [4,8,11,12].

The main purpose of this work was to investigate of photoactivity of Fe(III)-doped TiO_2_ nanoparticles in degradation of phenol under UV and visible light irradiation and then compared of results at the optimal condition (pH and catalyst dosage) with P25 TiO_2_ photoactivity under UV and visible light irradiation. The effects of various experimental parameters on photocatalytic degradation, such as pH, initial concentration of phenol and amount of photocatalyst were examined and optimized. Sol–gel method was selected to synthesis of Fe(III)-doped TiO_2_ nanoparticles due to its flexibility to control pore structures and dopant concentration, and high level of chemical purity [5]. Also efficiency of UV irradiation alone and amount of phenol adsorption on Fe(III)-doped TiO_2_ at dark condition was investigated.

## Materials and methods

### Preparation of the Fe(III)-doped TiO_2_ photocatalysts

121.775 mL absolute propanol and 62.77 mL TTIP were mixed and stirred for about 10 minutes. For adjusting pH of solution to 3, 2 mL nitric acid was added dropwise to the solution during 30 minutes, stirring was continued at long of this time (30 minutes). Then 8.33 mL double distilled water and 121.775 mL absolute propanol was vigorously stirred and added dropwise to the parent solution. For doped TiO_2_, Fe(NO_3_)_3_.9H_2_O were added to this solution and stirring continued for 90 minutes. For gel formation and exit of alcohol, the formed sol was stirred by use of a simple magnetic stirrer at room temperature for 24 h; after that the wet gel was dried under vacuum at 85°C for about 12 h and then calcined at 500 ± 50°C for 2–3 h [14].

### Characterization

The X–ray diffraction (XRD) patterns were obtained by a diffractometer (D8 Advanced Bruker AXS) with Cu Kα radiation. Carbon monochromator was used to determine the identity of each phase. A transmission electron microscope (TEM), (FEG Philips CM 200) was applied to observe the morphology of catalysts and estimate the particle size. The surface morphology was observed using a scanning electron microscope (SEM), (Model CamScan MV2300) equipped with an energy dispersive spectroscopy system (EDX, Oxford). In order to prevent the charge build–up during SEM observations, samples were coated with gold.

### Photoreactor

Photoactivity studies were conducted at the atmospheric pressure and room temperature (25°C). Photocatalytic degradation experiments were carried out in a 2 L Pyrex batch reactor of cylindrical shape (contained 1.5 L phenol solution). The reactor was placed in a box without any pore to prevent of entrance or exist of light from outside and inside. A 27 W low pressure lamp (Trojan) was used as the UV light source that was placed in a quartz jacket (50 mm inside diameter and 300 mm height) and submerged at the center of the cylindrical vessel to provide better irradiation. Visible light source was a 27 W lamp, that to making of similar condition, it also was placed in quartz jacket and submerged at the center of the cylindrical vessel. The distance between the light source and the bottom of the vessel was 1.5 cm. In order to assist the solution homogeneity, a simple magnetic stirrer was used. Phenol and all other chemicals were purchased from Merck Co. (Germany) and were of reagent grade quality.

Stock solution of phenol was first prepared according to directions outlined in Standard Methods [15]. At each experimental stage, 1.5 L solution containing phenol at designed concentration (5, 10, 50, 100 and 500 mg/L) was prepared by dilution of the stock solution with double distilled water; the experiment was then carried out as follows:

### Degradation of phenol by Fe(III)-doped TiO_2_/UV

In the first phase, photocatalytic degradation of up mentioned concentrations of phenol at three different pH (3, 7 and 11) and with three different concentrations of Fe(III)-doped TiO_2_ (0.25, 0.5 and 1 g/L) under UV irradiation was investigated. For all experiments pH was adjusted by NaOH (1 mol/L) and H_2_SO_4_ (0.1 mol/L). Before irradiation, the suspension was stirred continuously in dark for 30 min to ensure adsorption/desorption equilibrium. The irradiation time was 210 minutes and 10 mL of solution was withdrawn from the reactor after certain intervals (every 30 minutes). During the experiments the magnetic stirrer was employed to keep the suspensions uniform. Liquid samples were centrifuged at 6000 r/min for 10 min subsequently and filtered to separate Fe(III)-doped TiO_2_ particles. The concentration of phenol in the filtrates was measured using UV–vis spectrophotometer (Perkin-Elmer Lambda 25). The UV–vis spectrophotometer was set at a wavelength of 500 nm for analysis of phenol [15]. A quartz cell with a path length of 5 cm was used for spectrophotometric measurements. Based on the results of this stage, the optimum pH and photocatalyst concentration for phenol degradation were achieved. Also the effects of initial phenol concentration on degradation rate and photocatalytic degradation products of phenol under Fe(III)-doped TiO_2_/UV process were determined.

### Degradation of phenol by Fe(III)-doped TiO_2_/Vis

Photocatalytic degradation of all studied phenol concentrations at optimum conditions (pH and Fe(III)-doped TiO_2_ concentration) based on the results of former stage, were investigated under Visible light (27 W). The irradiation time was 210 minutes and similar to before stage, after certain intervals sampling was done.

### Degradation of phenol solely by UV irradiation

For this phase of the study, degradation of phenol at mentioned concentrations and the same pH (3, 7 and 11) was investigated under UV irradiation.

### Phenol adsorption of Fe(III)-doped TiO_2_

At this stage adsorption of phenol at 10 mg/L concentration on 0.5 g/L Fe(III)-doped TiO_2_ nanoparticles was examined at dark (the pHs level were the same as before).

### Degradation of phenol by TiO_2_/UV and Vis

In order to compare of photo activity of Fe(III)-doped TiO_2_ and TiO_2_ nanocatalysts under UV and Vis light, 10 mg/L of phenol at optimum conditions (pH and nanoparticles concentration) based on the results of first stage, was investigated.

## Results and discussion

### XRD, SEM EDX and TEM analysis

**Figure 1:**
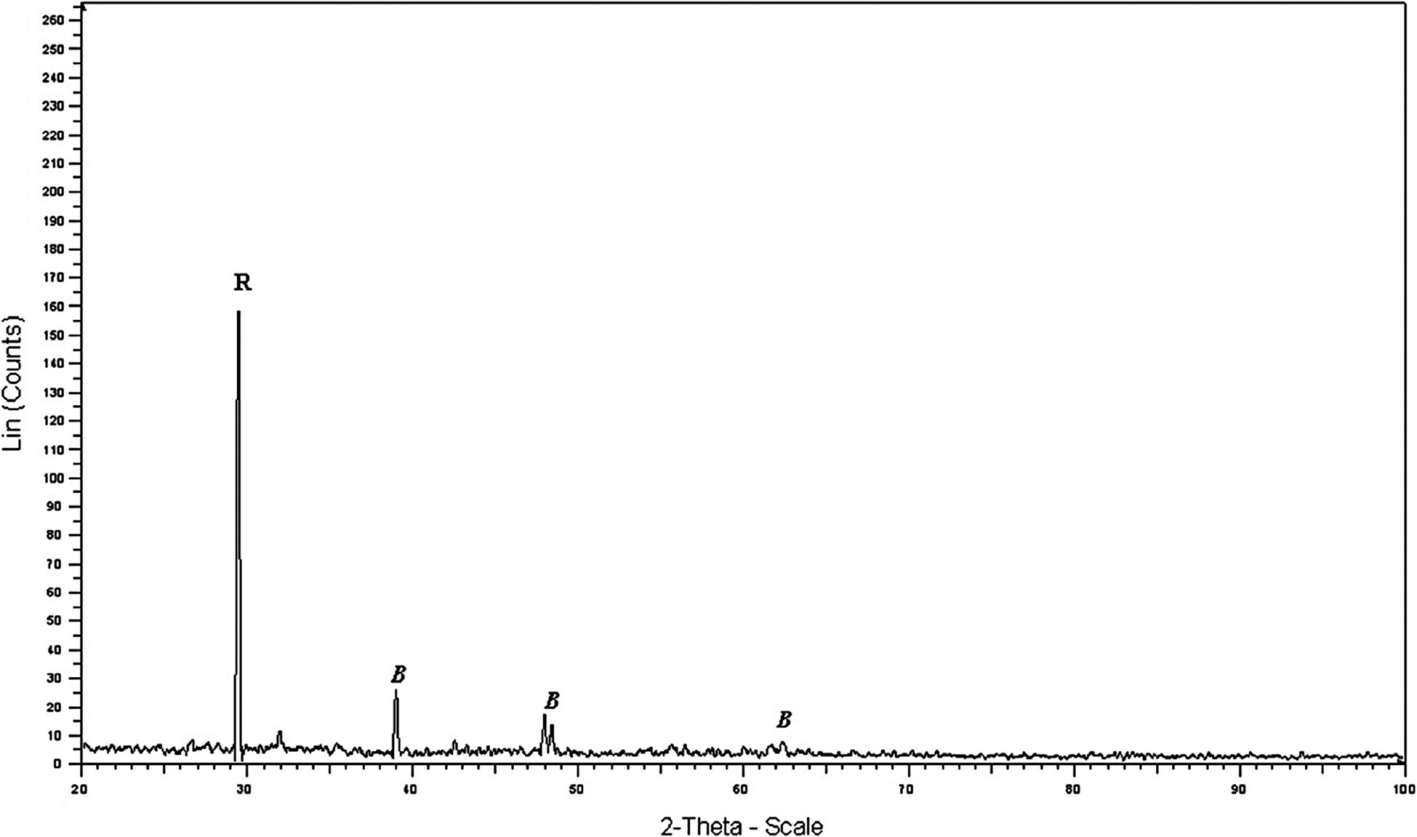
**XRD pattern of the Fe(III)-doped TiO**_**2 **_**sample; Fe/Ti = 0.034 at.%, calcination temperature: 500 ± 50°C (R: rutile; B: β-TiO**_**2**_**).**

**Figure 2:**
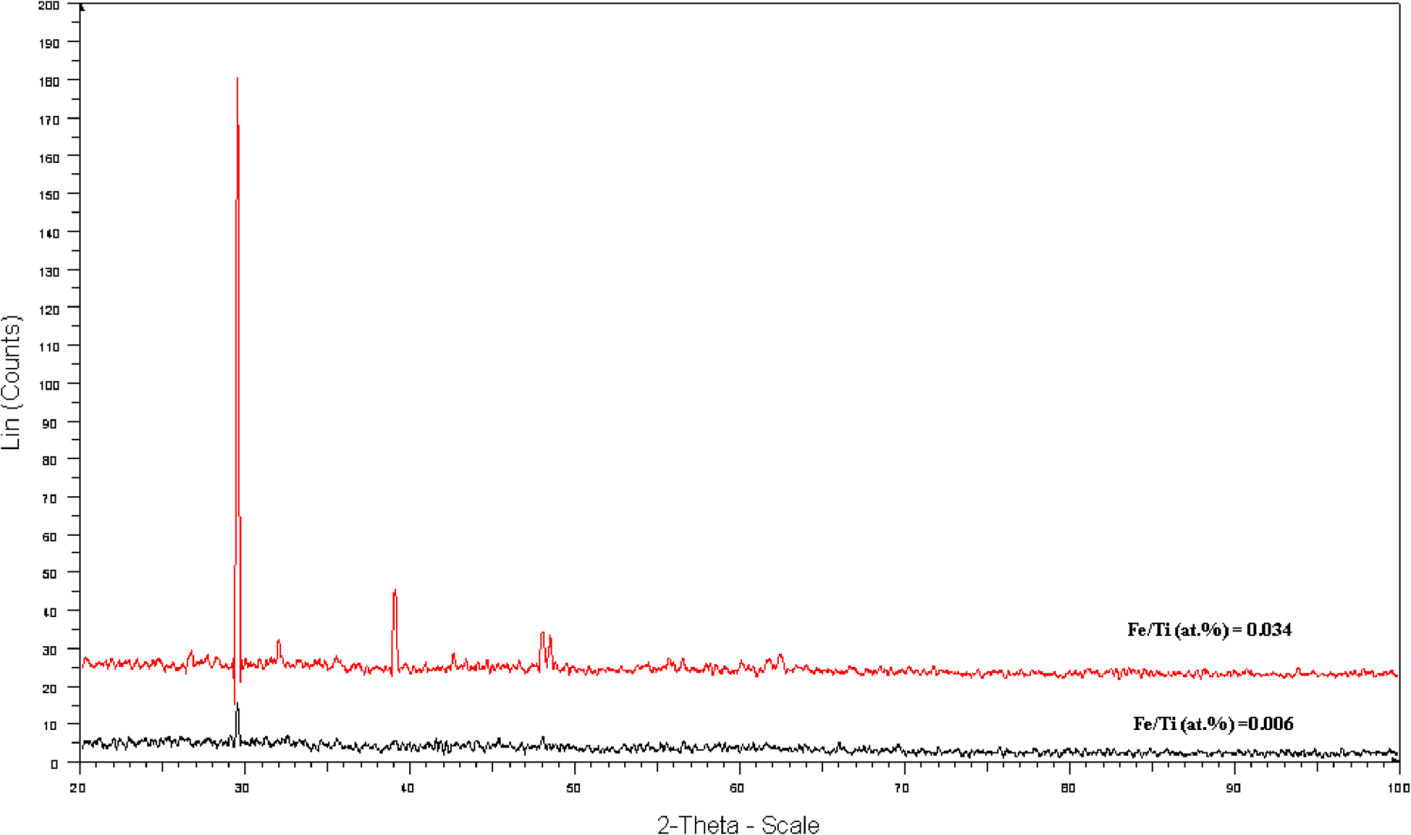
**Comparison between XRD patterns of Fe(III)-doped TiO**_**2 **_**nanopartcles at two atomic ratio (at.%) of Fe/ Ti = 0.034 and 0.006.**

Figure 1 depicts the XRD spectrum of the Fe (III)-doped TiO_2_ at atomic ratio of Fe to Ti, 0.034% prepared by the sol–gel method. In Figure 2, a comparison is made between XRD patterns of Fe(III)-doped TiO_2_ at two atomic ratio (at.%) of Fe/Ti = 0.034 and 0.006. The diffraction picks of the sample shows the presence of both rutile and β TiO_2_ or nanorod TiO_2_ phases. No hint of iron containing phases could be resolved in these diffractograms, which suggest that the amounts of Fe were low to be detected by XRD.

**Figure 3:**
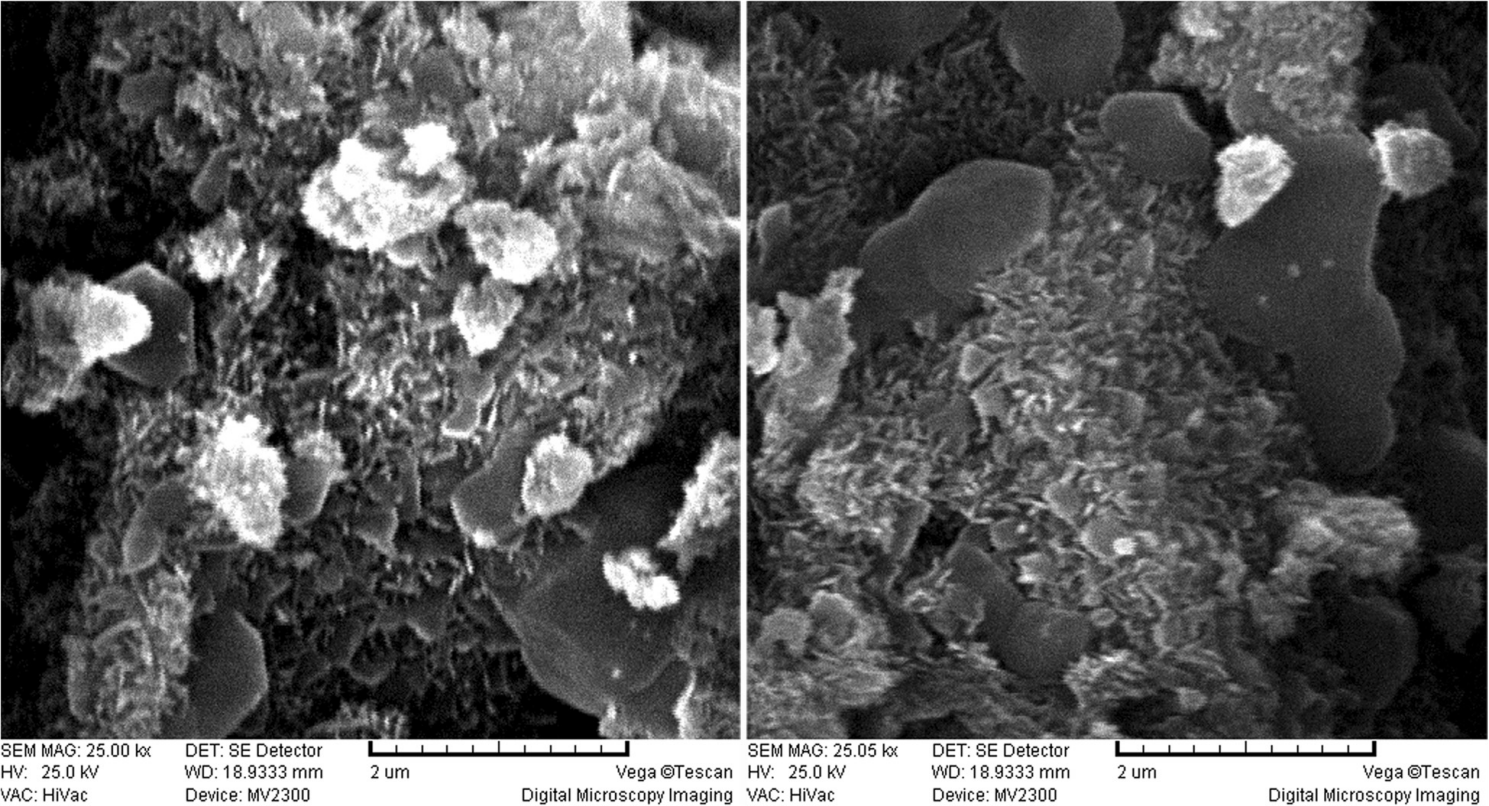
**SEM images of the Fe (III)-doped TiO**_**2 **_**sample from two different sides.**

**Figure 4:**
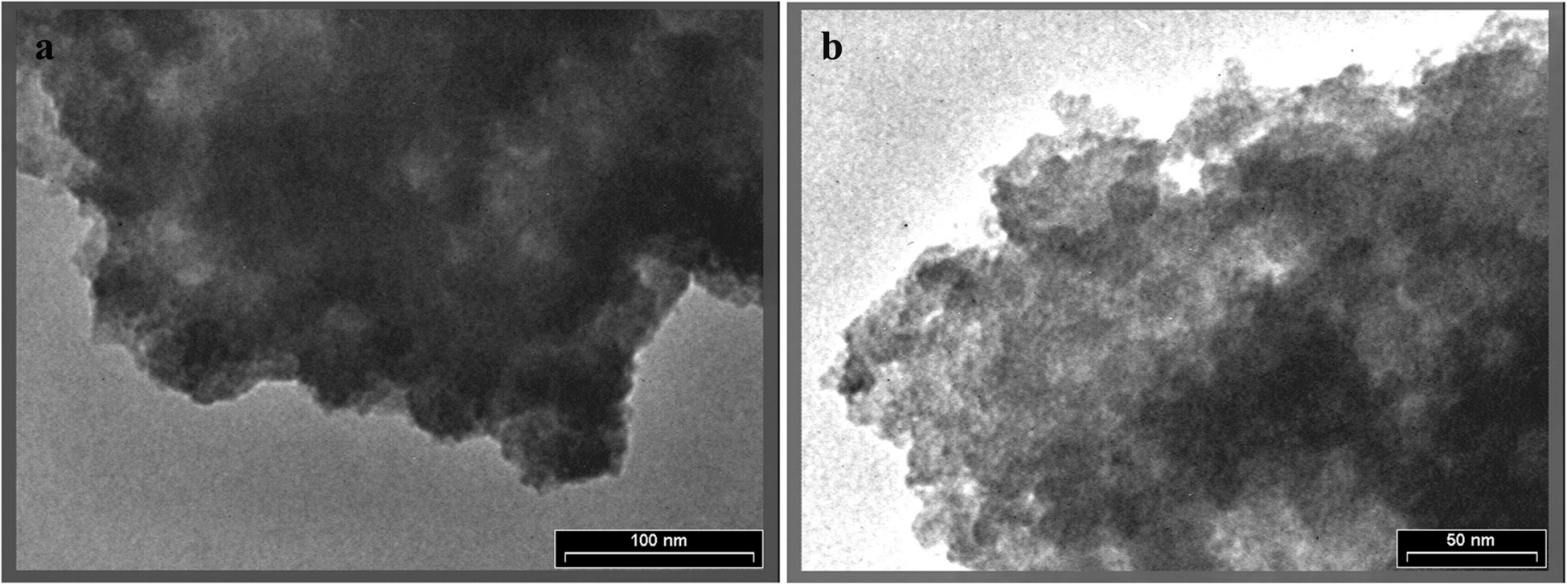
**TEM images of the Fe (III)-doped TiO**_**2 **_**sample (Scale bar=100 nm in panel a; scale bar=50 nm in panel b).**

The SEM images of Fe (III)-doped TiO_2_ nanoparticles are shown in Figure 3 which confirm the presence of β TiO_2_. The particle size distribution determined from SEM images was less than 50 nm. The atomic ratio of Fe to Ti, 0.034% was estimated from the EDX analysis. TEM results (Figure 4 (a and b)) revealed that the sample consisted of agglomerates of particles 10–50 nm in size, which is in general agreement with the SEM findings.

### Effect of initial phenol concentration

**Figure 5:**
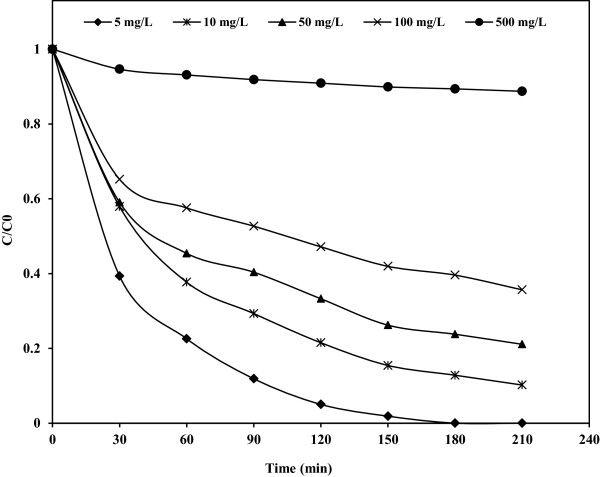
**Effect of initial concentration of phenol on the photocatalytic degradation of phenol.** Fe(III)-doped TiO_2_ = 0.5 g/L, pH = 3.

It is well known that the initial concentration of reactant plays an important role on photodegradation of organic compounds [16]. The influence of initial phenol concentration on photocatalytic degradation at five levels (5, 10, 50, 100 and 500 mg/L) was investigated (Figure 5). As shown in figure, photocatalytic degradation decreases with increasing initial concentration. Decrease in degradation rate at higher concentration is attributed the fact that light absorbed by the phenol is more than that of Fe(III)-doped TiO_2_. Thus light absorbed is not effective to carry out the degradation [6,17]. Further, the equilibrium adsorption of phenol on the catalyst surface active site increases and more and more molecules of phenol are adsorbed by the catalyst [6,17,18]. As a result, competitive adsorption of OH^−^ on the same site decreases and consequently the amount of ^•^OH and O_2_^•−^ on the surface of catalyst decreases. For all initial phenol concentrations, the catalyst dosage, irradiation time and intensity of light were constant. Since the generation of ^•^OH does not increase, the probability of phenol molecules to react with ^•^OH decreases and hence, a decrease in the degradation efficiency is observed [17]. In fact, with progress in degradation reaction especially at high initial concentration, some intermediates are formed and competitively adsorbed on the catalyst surface and also competitively react with oxidant species [18-20]. Moreover, the oxidized intermediate can react with reducing species (e.g. electrons) yielding back phenol which finally results in a decrease of the degradation rate of the substrate [21].

### Effect of pH

**Figure 6:**
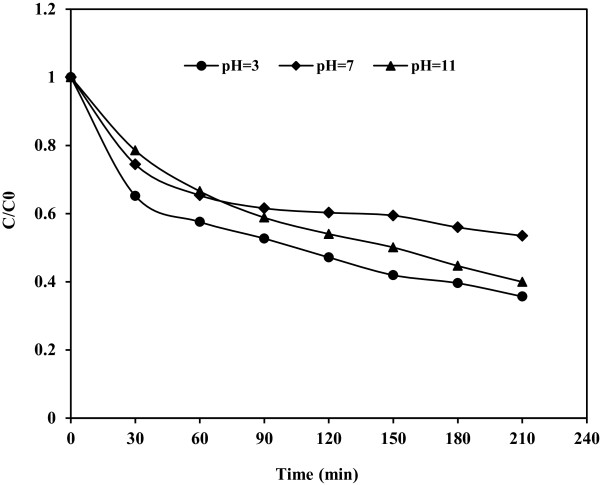
**Effect of pH on photocatalytic degradation of phenol; C**_**0 **_**= 100 mg/L and Fe(III)-doped TiO**_**2 **_**= 0.5 g/L.**

Figure 6. illustrates the effect of pH on phenol degradation (C/C_0_, where C_0_ is the initial phenol concentration and C is the phenol concentration at time t). The highest degradation efficiency occurred at pH = 3 and the lowest degradation occurred at pH = 7. This is attributed to the fact that, condition in addition to OH radicals produced by Fe(III)-doped TiO_2_/UV process, there are more hydrogen ions at acidic condition and these ions can cause the production of more OH radicals (as a major agent of degradation at photocatalytic reactions) to degrade phenol. This conclusion is similar to the report of Guo et al. [21], which indicated that the H^+^ ions have important role on OH radicals formation. But higher phenol degradation at pH = 11 in comparison with neutral pH is due to the presence of phenol molecules as negatively charged phenolate species. These anions are more reactive than phenol molecules. Also in alkaline conditions there is an increase in the concentration of OH radicals [22]. Although this increase can be cause of more degradation of phenol at alkaline pH in comparison with neutral pH, but when the concentration of OH^−^ is higher in the solution, it prevents the penetration of UV light to reach the catalyst surface. Moreover, high pH favors the formation of carbonate ions which are effective scavengers of OH^−^ ions and can reduce the degradation rate [17,23]. These can be cause of the less degradation of phenol at alkaline pH in comparison with acidic pH.

### Effect of catalyst dosage

**Figure 7:**
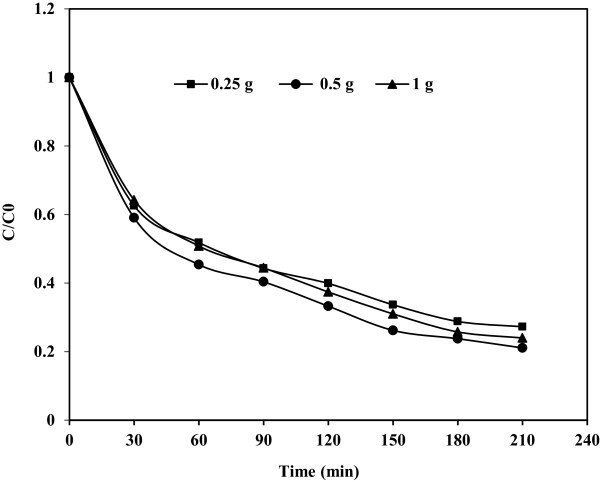
**Effect of catalyst dosage on photocatalytic degradation of phenol; C**_**0 **_**= 50 mg/L and pH = 3.**

In slurry photocatalytic processes, catalyst dosage is an important parameter that has been extensively studied. Figure 7. shows the influence of the catalyst concentration on photocatalytic degradation of phenol. As expected with the increase in concentration of catalyst from 0.25 to 0.5 g/L, degradation of phenol increases. According to some of investigations [6,17], this is due to the fact that the increase in the number of Fe(III)-doped TiO_2_ particles will increase the number of photons absorbed, the available active sites and consequently the number of the phenol molecule adsorbed. But there was not a considerable increase in phenol degradation when catalyst concentration was increased to 1 g/L. This is attributed to the fact that, agglomeration and sedimentation of it under large catalyst loadings would also take place and available catalyst surface for photon absorption would actually decrease. In fact, the opacity and screening effect of excess Fe(III)-doped TiO_2_ act as a shield, and consequently hinder the light penetration, causing available surface area loss for light-harvesting and reduction of the catalytic activity, as reported earlier [6,17,21,24,25]. Therefore, the optimal dosage of Fe(III)-doped TiO_2_ was determined as 0.5 g/L.

### Photoactivity comparison

**Figure 8:**
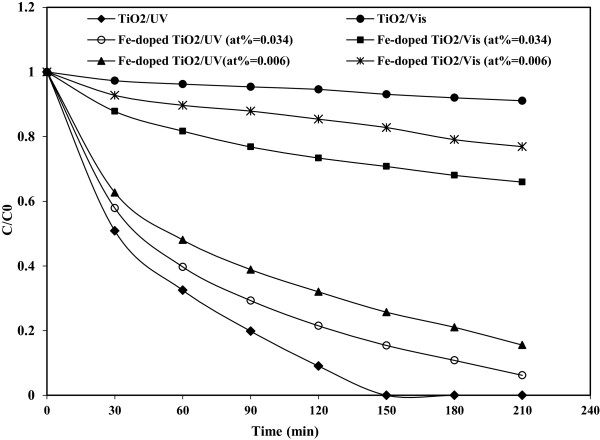
**Comparison of photoactivty of Fe(III)-doped TiO**_**2 **_**and P25 TiO**_**2 **_**nanoparticles under UV and visible light.**

Figure 8 shows comparison of photoactivty of Fe(III)-doped TiO_2_ nanoparticles with two different content of Fe and P25 TiO_2_ nanoparticles under UV irradiation and visible light on photocatalytic degradation of phenol at optimum condition (pH = 3, catalyst dosage = 0.5 g/L). As shown in the figure, the degradation rate of phenol under Fe(III)-doped TiO_2_/Vis was higher than degradation rate under TiO_2_/Vis. This observation confirms that Fe(III) ions play an improvement role in TiO_2_ structure and increases activity of TiO_2_ to visible light.

Also figure shows that degradation decreased at atomic ratio of Fe/Ti, 0.006% in compared with Fe/Ti atomic ratio, 0.034%. Whereas the Iron ions at TiO_2_ lattice can act as both electron and hole traps to reduce the recombination rate and this can increase photocatalytic activity. Therefore the decrease of photoactivity of Fe(III)-doped TiO_2_ with the decrease of Fe content can be due to the increase of recombination rate of photogenerated electron–hole pairs and also the decreasing of available trapping sites. The study of Hu et al. [9] also indicated that the amount of Fe is very important at photoactivity of Fe(III)-doped TiO_2_ and high or low level of doping decreases the photocatalytic activity of Fe(III)-doped TiO_2_.

This figure indicated that the efficiency of phenol degradation at optimum conditions under TiO_2_/UV process was higher in comparison with Fe(III)-doped TiO_2_/UV. It can be due to this fact that TiO_2_ particles are smaller and more uniform than Fe(III)-doped TiO_2_ particles. Also high efficiency of Fe(III)-doped TiO_2_ under UV irradiation in degradation of phenol compared with Fe(III)-doped TiO_2_ under visible light suggests that the excitation energy of the UV is higher than visible light to transit electrons of the valence band to the conduction band. This result is consistent with the results of Shamsun Nahar et al. [26] who reported that UV activity was several times higher than that under visible light irradiation.

**Figure 9:**
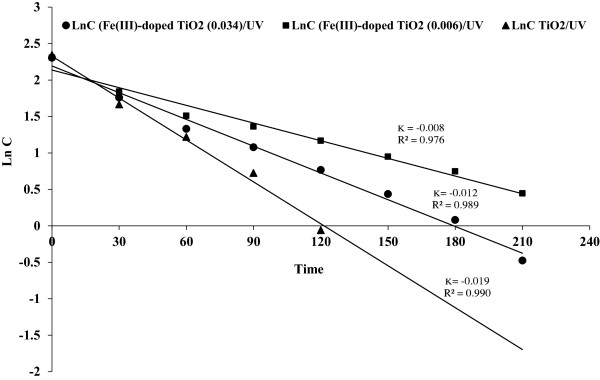
**Pseudo first-order degradation rate of phenol (Fe(III) doped TiO**_**2**_**/UV and TiO**_**2**_**/UV processes).**

Besides results indicated that all calculated values of -ln (C/C_o_) (C_o_ is the initial phenol concentration and C is phenol concentration at time t) in degradation of phenol under both Fe(III)-doped TiO_2_/UV and P25 TiO_2_/UV processes follows a linear model with the elapse of irradiation time. This means that the pseudo first order kinetics relative to phenol is operative (Figure 9).

**Figure 10:**
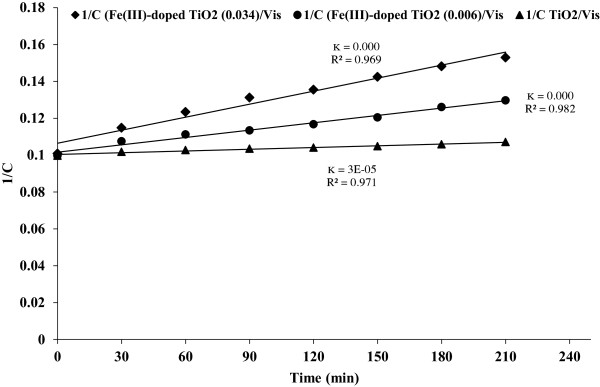
**Pseudo second-order degradation rate of phenol (Fe(III)- doped TiO**_**2**_**/Vis and TiO**_**2**_**/Vis processes).**

However according to the results the degradation behavior of phenol by Fe(III)-doped TiO_2_ and P25 TiO_2_ under visible light obeys pseudo second order kinetics (Figure 10).

k_app_ values (the apparent kinetic or apparent rate constant (min^−1^ in pseudo first order and Lmg^−1^ min^−1^ in pseudo second order) and correlation coefficients for phenol oxidation are given in the figures. As observed in the Figure 9, k_app_ increases with increasing of degradation rate (TiO_2_ > Fe(III)-doped TiO_2_ (0.034) > Fe(III)-doped TiO_2_ (0.006)).

### Effect of UV irradiation

Investigation of phenol degradation under solely UV irradiation showed that the degradation rate of phenol was low in comparison with using catalyst during 210 minutes. Also phenol adsorption on Fe(III)-doped TiO_2_ specimen displayed that at these conditions the changes of concentration was negligible (Table 1).

**Table 1:** **Removal efficiency of phenol by Fe-doped TiO**_**2**_**/UV, solely UV irradiation and Fe-doped TiO**_**2 **_**adsorption (percent)**

**Fe-doped TiO**_**2 **_**adsorption**	**Solely UV irradiation**	**Fe-doped TiO**_**2**_**/UV**	**Time (min)**
0.27	24.74	42.12	30
1.58	40	60.28	60
2.31	48.8	70.7	90
3	55.6	78.51	120
4	59.43	84.57	150
4.5	62	89.2	180
5.2	64.8	93.8	210

## Conclusion

Photocatalytic degradation of phenol has been carried out over Fe(III)-doped TiO_2_ (prepared by sol–gel method) and P25 TiO_2_ under UV irradiation and visible light. Also Effect of pH, catalyst dosage, initial phenol concentration, UV irradiation on degradation efficiency was investigated. Results showed that at appropriate atomic ratio of Fe to Ti (% 0.034) photoactivity of Fe(III)-doped TiO_2_ nanoparticles increased. At all different initial concentration, highest degradation efficiency occurred at pH = 3 and 0.5 g/L Fe(III)-doped TiO_2_ dosage. Experimental results showed that the degradation rate decreased with an increase in the initial concentration of phenol. Also photoactivity comparison showed that the photoactivity of Fe(III)-doped TiO_2_ nanoparticles under visible light was higher than P25 TiO_2_ particles. However experimental results showed that the P25 TiO_2_ nanoparticles under UV irradiation had higher efficiency for phenol degradation in comparison with Fe(III)-doped TiO_2_/UV process. According to the results concentration of Fe(III) ions in doping process has important role in photoactivity of Fe(III)-doped TiO_2_ nanoparticles. Photocatalytic degradation of phenol by Fe(III)-doped TiO_2_ and P25 TiO_2_ nanoparticles under UV irradiation and visible light obey pseudo first order and pseudo second order kinetics subsequently. Also degradation rate under solely UV irradiation was lower in comparison with situations that catalyst was used, and adsorption of phenol on the Fe(III)-doped TiO_2_ was negligible at dark.

## Competing interests

The authors declare that they have no competing interests.

## Authors’ contributions

SHB carried out all the labworks (experiments and nanoparticles synthesis) under the guidance of SN, AHM and RN AHJ. RN also contributed in analyzing of data. AHJ contributed in synthesis of nanoparticles. AHM contributed in reviewing of the manuscript. The overall implementation of this study carried out under the guidance of SN. All authors read and approved the final manuscript.
